# Vitamin K metabolism as the potential missing link between lung damage and thromboembolism in Coronavirus disease 2019

**DOI:** 10.1017/S0007114520003979

**Published:** 2020-10-07

**Authors:** Rob Janssen, Margot P. J. Visser, Anton S. M. Dofferhoff, Cees Vermeer, Wim Janssens, Jona Walk

**Affiliations:** 1 Department of Pulmonary Medicine, Canisius-Wilhelmina Hospital, 6532 SZ Nijmegen, The Netherlands; 2 Department of Internal Medicine, Canisius-Wilhelmina Hospital, 6532 SZ Nijmegen, The Netherlands; 3 Department of Biochemistry, Cardiovascular Research Institute Maastricht, Maastricht University, 6229 ER Maastricht, The Netherlands; 4 Department of Respiratory Diseases, University Hospitals Leuven, 3000 Leuven, Belgium

**Keywords:** Vitamin K, Covid-19, Matrix Gla protein, Protein S, Prothrombin

## Abstract

Coronavirus disease 2019 (Covid-19), caused by severe acute respiratory syndrome coronavirus (SARS-CoV)-2, exerts far-reaching effects on public health and socio-economic welfare. The majority of infected individuals have mild to moderate symptoms, but a significant proportion develops respiratory failure due to pneumonia. Thrombosis is another frequent manifestation of Covid-19 that contributes to poor outcomes. Vitamin K plays a crucial role in the activation of both pro- and anticlotting factors in the liver and the activation of extrahepatically synthesised protein S which seems to be important in local thrombosis prevention. However, the role of vitamin K extends beyond coagulation. Matrix Gla protein (MGP) is a vitamin K-dependent inhibitor of soft tissue calcification and elastic fibre degradation. Severe extrahepatic vitamin K insufficiency was recently demonstrated in Covid-19 patients, with high inactive MGP levels correlating with elastic fibre degradation rates. This suggests that insufficient vitamin K-dependent MGP activation leaves elastic fibres unprotected against SARS-CoV-2-induced proteolysis. In contrast to MGP, Covid-19 patients have normal levels of activated factor II, in line with previous observations that vitamin K is preferentially transported to the liver for activation of procoagulant factors. We therefore expect that vitamin K-dependent endothelial protein S activation is also compromised, which would be compatible with enhanced thrombogenicity. Taking these data together, we propose a mechanism of pneumonia-induced vitamin K depletion, leading to a decrease in activated MGP and protein S, aggravating pulmonary damage and coagulopathy, respectively. Intervention trials should be conducted to assess whether vitamin K administration plays a role in the prevention and treatment of severe Covid-19.

## Graphical abstract

**Figure:**
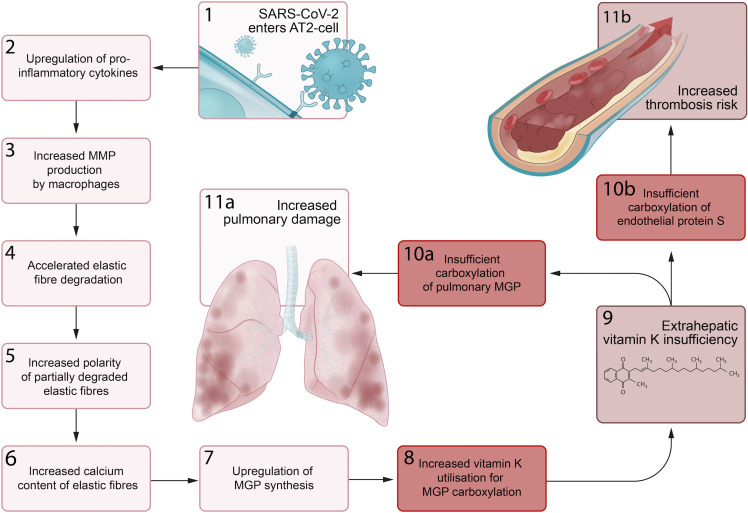

Coronavirus disease 2019 (Covid-19) is an infectious disorder caused by the severe acute respiratory syndrome coronavirus (SARS-CoV)-2 that emerged from the Chinese city of Wuhan at the end of 2019 and has since then relentlessly spread across the globe^(1)^. The Covid-19 pandemic is causing a worldwide medical and socio-economic crisis of unprecedented proportions in modern times. The vast majority of individuals who contract SARS-CoV-2 have mild to moderate symptoms. However, a significant minority develops respiratory failure due to pneumonia and/or acute respiratory distress syndrome^(1)^.

Particularly, SARS-CoV-2-infected individuals suffering from certain premorbid conditions, such as hypertension, diabetes, CVD and obesity, are at increased risk of complicated disease course^(1)^. Although these conditions are also associated with poor outcomes due to other infectious diseases, precisely why these groups have high morbidity and mortality is currently unknown. We made the observation that these disorders are associated with elastic fibre pathologies as well as vitamin K insufficiency.

Thromboembolism and generalised microvascular thrombosis are also prevalent in severe Covid-19^(2,3)^. The mechanisms leading from pulmonary infection to systemic coagulopathy in Covid-19 have not yet been entirely elucidated. It has previously been shown that severe vitamin K deficiency in critically ill patients can be misdiagnosed as disseminated intravascular coagulation^(4)^. Given the importance of vitamin K-dependent proteins in coagulation as well as elastic fibre metabolism, we recently hypothesised that vitamin K is implicated in Covid-19 pathogenesis and could represent the missing link between pulmonary damage and thrombogenicity.

## Vitamin K metabolism

Vitamin K is a monofunctional nutrient from a biochemical perspective as its only well-described function is facilitating γ-carboxylation. However, it can be regarded as pleiotropic because it activates proteins with distinct, opposing and not yet fully unravelled functions.

Vitamin K catalyses the carboxylation reaction that transforms glutamic acid into γ-carboxyglutamic (Gla) residues and is well known as an activator of hepatic procoagulant factors II (prothrombin), VII, IX and X. However, vitamin K also activates anticoagulant proteins C and S as well as a number of extrahepatic proteins not involved in blood coagulation.

### Endothelial protein S

Contrary to other vitamin K-dependent procoagulant factors and protein C, which are almost exclusively hepatic proteins, about 50 % of anticoagulant protein S is produced outside the liver^(5)^. This part of protein S is mainly synthesised in endothelial cells and thought to play an important role in the local prevention of thrombosis^(5–7)^. Endothelium-produced protein S has the ability to associate with the cell surface and promote procoagulant factor V inactivation in the presence of activated protein C^(7)^.

### Matrix Gla protein

Vitamin K-dependent matrix Gla protein (MGP) has been extensively studied as an inhibitor of vascular mineralisation^(8)^; however, its role in the pulmonary compartment seems to be comparable^(9)^. Besides preventing soft tissue calcification, MGP also protects against elastic fibre degradation. This was demonstrated in MGP knockout mice, which developed severely mineralised as well as fragmented elastic fibres^(10)^.

Elastic fibres are critical components in the extracellular matrix of dynamic tissues^(11)^. They provide deformability to lungs and arteries, which facilitates respiration and circulation^(11)^. Initial elastic fibre development is almost exclusively restricted to the perinatal period^(11)^. Elastic fibre degradation and repair, however, are continuous processes^(11)^. The balance between the two is delicate and of vital importance for cardiovascular and pulmonary health^(12)^. The rate of proteolytic elastic fibre degradation increases during ageing^(13)^. This age-related acceleration of elastolysis is enhanced in certain pulmonary conditions such as chronic obstructive pulmonary disease and idiopathic pulmonary fibrosis^(13,14)^.

Affinity of elastic fibres for Ca is high^(15)^. Critically, elastic fibre calcification and proteolytic degradation processes are closely related. Partially degraded elastic fibres are more negatively charged, attracting positively charged Ca^(15)^. As elastic fibre Ca content increases, the synthesis of matrix metalloproteinases, proteolytic enzymes that degrade elastin fibres, is also up-regulated^(16)^. Peri-arterial application of Ca on rat abdominal aortas induces both calcification and proteolytic degradation of elastic fibres^(17)^. Subdermal implantation of elastin in rats results in significant calcification, but local application of a protease inhibitor attenuates this mineralisation^(18,19)^. MGP plays a critical role in the protection of elastic tissues against mineralisation^(10)^, most likely because other proteins that inhibit calcification (e.g. fetuin-A) are too large to enter the lumen of the fibres^(8,20)^.

### Vitamin K recycling

**Fig. 1.**
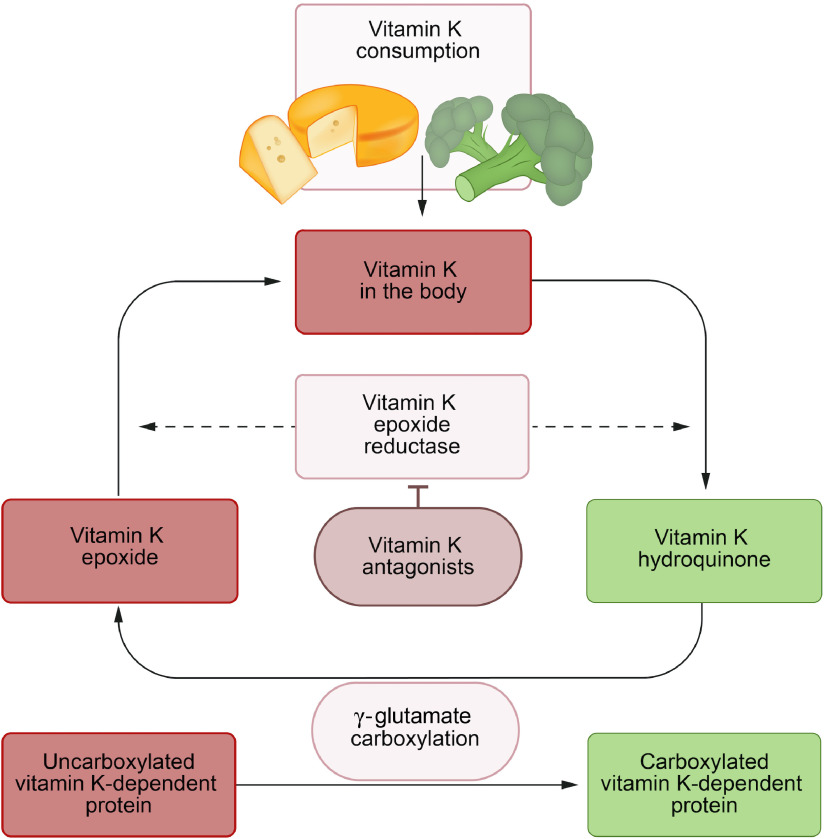
Vitamin K cycle. The vitamin K cycle and the effects of vitamin K antagonists. 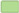, Active; 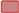, inactive.

Storage capacity of vitamin K is limited, and therefore, its metabolism must be very efficient. After being oxidised during the carboxylation reaction, vitamin K is reactivated repeatedly by the enzyme vitamin K epoxide reductase in the vitamin K cycle (Fig. 1)^(21)^. Nevertheless, insufficiency may develop within days of poor intake, particularly in pathological states of increased vitamin K utilisation^(4,9,22)^.

### Triage-based distribution

**Fig. 2.**
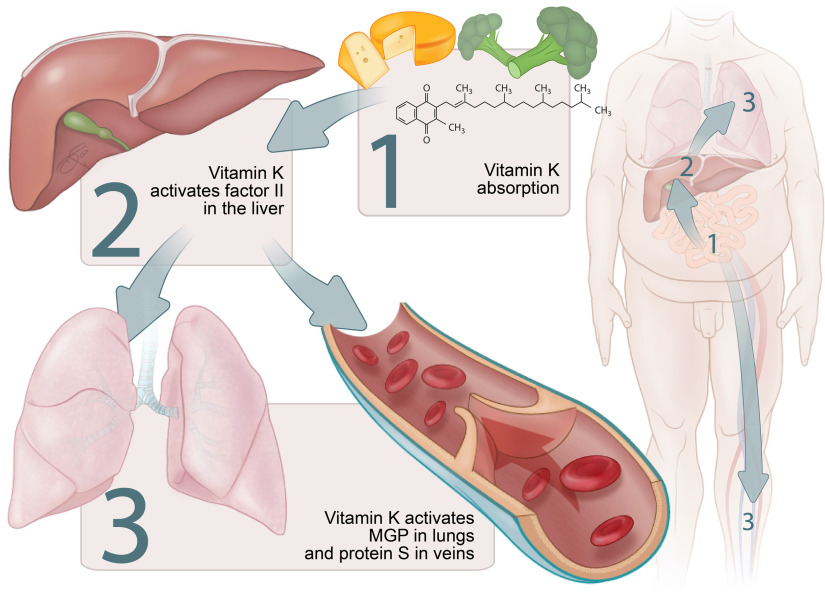
Micronutrient triage theory with regard to vitamin K. Particularly vitamin K_1_ is preferentially transported to the liver. This implies that the grade of carboxylation in a state of vitamin K deficiency is usually higher for hepatic procoagulant factors, such as factor II, than for endothelial protein S as well as for pulmonary matrix Gla protein (MGP).

The triage theory posits that during times of scarcity, micronutrients are reserved for use in processes that form the greatest threat to short-term survival if not properly executed^(23)^. This implies that in case of vitamin K insufficiency, the vitamin is preferentially transported to the liver for the activation of the above-mentioned procoagulant factors at the expense of extrahepatic vitamin K-dependent proteins such as MGP (Fig. 2).

This was demonstrated in women between 60 and 80 years old who consumed a vitamin K_1_-deficient diet for 28 d. Undercarboxylated osteocalcin, a vitamin K-dependent bone protein, increased almost immediately, whereas undercarboxylated factor II increased more slowly^(24)^. Moreover, when patients using vitamin K antagonists (VKA) as anticoagulants steadily increased their dietary intake of vitamin K_1_, a significant decrease in undercarboxylated factor II was seen at 150 μg/d, while a significant decrease in undercarboxylated osteocalcin was only seen at an intake of 300 μg/d^(25)^.

Similar to osteocalcin and MGP, vitamin K insufficiency would result in deficient activation of endothelial protein S before causing a decrease in carboxylated procoagulant factors (Fig. 2)^(26)^. This could explain the seemingly paradoxical increase of thrombosis risk in the first week of treatment with VKA^(27)^.

Although the biological function of vitamins K_1_ and K_2_ is similar, there are differences with regard to bioavailability and tissue distribution. Half-life times of most K_2_ vitamins are longer than that of K_1_, and vitamin K_2_ may have more extrahepatic potential than K_1_^(28)^. Vitamin K_1_ is found in green vegetables such as broccoli, spinach and kale. Certain bacteria have the ability to produce vitamin K_2_. It is therefore present in fermented food products such as cheese, curd and sauerkraut as well as in certain fishes.

### Assessment of vitamin K status

Measuring circulating levels of the two naturally occurring forms of vitamin K – vitamin K_1_ (phylloquinone) and K_2_ (the group of menaquinones) – is technically feasible^(29)^. However, the value of such measurements for assessing general vitamin K status is limited. Quantification of vitamin K-dependent proteins that have not been carboxylated, on the other hand, is a valuable method reflecting the combined functional deficit of vitamin K_1_ and K_2_^(29)^. Determination of desphospho-uncarboxylated (dp-uc; i.e. inactive) MGP levels and the ratio between uncarboxylated and carboxylated osteocalcin are validated assays of extrahepatic vitamin K status^(29)^.

High dp-ucMGP reflects low vitamin K status and *vice versa*. Although increasing vitamin K consumption decreases dp-ucMGP^(30–32)^, its levels are not simply a biomarker of vitamin K intake but depend on other factors as well. Circulating dp-ucMGP concentration can best be regarded as a reflection of the total extrahepatic vitamin K deficit, that is, the amount of vitamin K that is needed to carboxylate all the uncarboxylated vitamin K-dependent proteins in the body^(33)^.

Hepatic vitamin K status is usually quantified by measuring levels of protein induced by vitamin K absence (PIVKA)-II (i.e. uncarboxylated prothrombin)^(24)^.

## Vitamin K metabolism in Coronavirus disease 2019

Extrahepatic vitamin K status is severely reduced in Covid-19 patients, reflected by elevated dp-ucMGP levels^(34)^. Reasons for this could include premorbid low vitamin K status in combination with accelerated utilisation during infection.

### Vitamin K status in co-morbidities associated with poor Coronavirus disease 2019 outcomes

dp-ucMGP levels are elevated in various diseases that are associated with elastic fibre calcification and degradation such as diabetes^(35)^, hypertension^(36)^, CVD^(37)^, chronic kidney disease^(35,38)^ and obesity^(39)^. It is possible that reduced vitamin K intake increases the risk of developing these conditions^(40)^. However, increased vitamin K demand due to enhanced utilisation may be another important cause of high dp-ucMGP^(41)^. Partially degraded and mineralised elastic fibres – which are prevalent in diabetic, hypertensive, renal and cardiovascular patients – are more vulnerable to further proteolysis and calcification^(17,42)^. This increases the need for MGP synthesis to protect elastic fibres^(9)^, draining vitamin K stores for MGP carboxylation and leading to higher dp-ucMGP levels.

In vascular diseases^(40)^, as well as in the general population^(43,44)^, increased dp-ucMGP levels associate with higher all-cause mortality. Vitamin K supplementation reduces dp-ucMGP levels and has a favourable effect on progression of clinically relevant endpoints, including aortic valve calcification, arterial stiffness and bone loss^(30–32)^.

### Vitamin K insufficiency in the pathogenesis of Coronavirus disease 2019

**Fig. 3.**
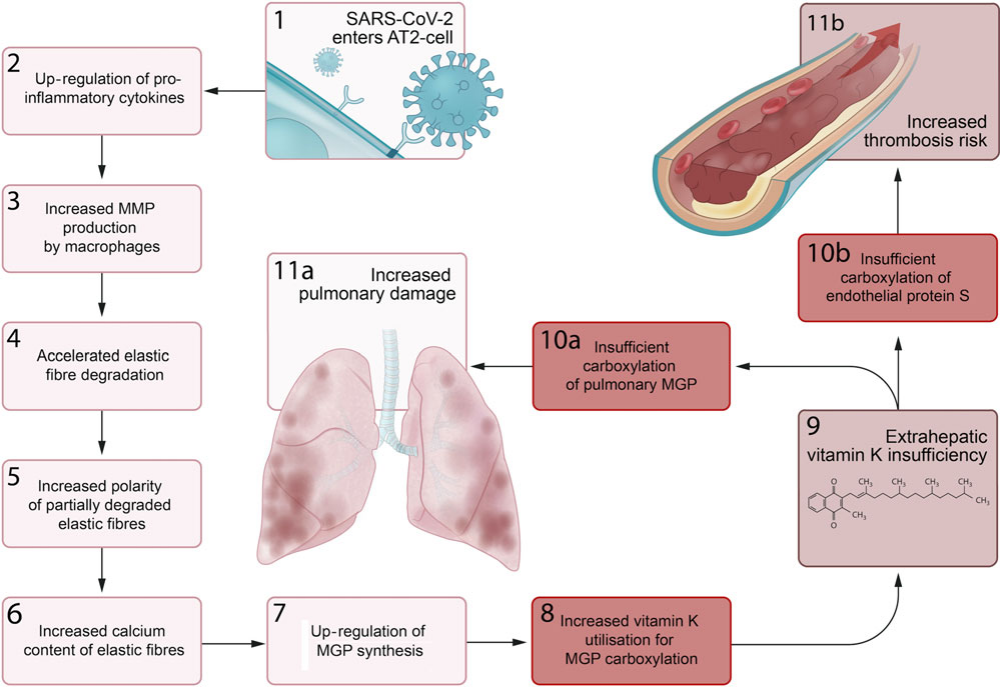
Proposed sequential steps linking severe acute respiratory syndrome coronavirus-2 (SARS-CoV-2) pneumonia to vitamin K insufficiency, pulmonary damage and thrombogenecity. (1) SARS-CoV-2 enters alveolar type II (ATII) cells. (2) Infected AT2 cells response by up-regulating synthesis of proinflammatory cytokines, including IL-6. (3) This leads to an increase in the number and activation of alveolar macrophages (4) that produce matrix metalloproteinases (MMP), which accelerates degradation of elastic fibres. (5) The increased polarity of partially degraded elastic fibres (6) enhances their affinity for calcium and leads to increased elastic fibre calcium content. (7) Matrix Gla protein (MGP) synthesis is up-regulated in an attempt to protect elastic fibres from calcification and degradation, (8) and the need for vitamin K to carboxylate additional MGP increases. (9) This increased utilisation of vitamin K may induce extrahepatic vitamin K insufficiency, (10a) leading to insufficient carboxylation of pulmonary MGP and (11a) increased pulmonary damage. (10b) The second consequence of extrahepatic vitamin K insufficiency is decreased carboxylation of endothelial protein S, (11b) which increases thrombosis risk.

We propose a series of sequential pathological steps occurring in response to SARS-CoV-2 infection that are responsible for up-regulation of MGP expression and extrahepatic vitamin K depletion, leading to pulmonary damage and thrombosis in Covid-19 (Fig. 3).

Infection primarily begins through the inhalation of SARS-CoV-2-containing aerosols. The virus’ so-called ‘spike proteins’ have the ability of binding to angiotensin-converting enzyme 2 receptors, which enables viral entry into human cells, including alveolar type II cells. These proteins are also the most immunogenic part of the virus^(45)^.

Although Covid-19 is a communicable disease, morbidity and mortality are mainly attributable to immunological rather than direct infectious complications. SARS-CoV-2 has the ability to put the immune system of infected individuals into overdrive resulting in a hyperinflammatory pulmonary state^(46)^. Data from SARS infections suggest that the synthesis of proinflammatory cytokines begins in infected alveolar type II cells^(47)^. Increased levels of IL-6 and TNF-α are associated with poor Covid-19 outcome^(48)^. Autopsy studies consistently implicate infiltrating lymphocytes and macrophages as further drivers of pulmonary inflammation during Covid-19^(49,50)^. Importantly, a specific subset of macrophages appears in the lungs of Covid-19 patients^(51)^, which has previously been demonstrated to produce matrix metalloproteinases-9 in patients with idiopathic pulmonary fibrosis^(52)^.

Upon SARS-CoV-2 infection, elastic fibre breakdown accelerates compared with degradation rates in age-matched controls^(34)^. High pulmonary concentrations of matrix metalloproteinases-9 or other proteases produced either by infiltrating or locally proliferating macrophages could be an explanation for this^(51,52)^. A significant correlation between dp-ucMGP and the rate of elastic fibre degradation was observed in Covid-19 patients^(34)^. We suspect that accelerated elastic fibre degradation due to enhanced proteolytic activity in SARS-CoV-2-infected lungs increases elastic fibre vulnerability to Ca, leading to an up-regulation of MGP synthesis and depletion of extrahepatic vitamin K stores. This shortage impairs MGP activation, presumably causing further elastic fibre damage, and elevation of circulating dp-ucMGP. The rate of elastic fibre degradation associates with poor outcome in patients with SARS-CoV-2 pneumonia^(34)^, as it does in other pulmonary diseases such as chronic obstructive pulmonary disease, cystic fibrosis and bronchiectasis^(13,53,54)^.

Conditions associated with chronic elastic fibre pathology, including diabetes, hypertension and CVD, are also related to worse prognosis of SARS-CoV-2 infection. Recent data demonstrated that Covid-19 patients with poor outcomes had increased thoracic aortic and coronary artery calcification on computed tomography scan, though these analyses lost significance after correction for age and sex^(34)^. Nevertheless, pre-existing elastic fibre damage predisposes to enhanced proteolytic degradation during inflammation^(42)^, potentially explaining the increased severity of Covid-19 in those populations.

We further theorise that vitamin K depletion also has a key effect on another characteristic disease manifestation of Covid-19. Though dp-ucMGP is elevated in Covid-19, hepatic procoagulant vitamin K status, quantified by measuring PIVKA-II, was hardly affected^(34)^. According to the micronutrient triage theory^(23)^, the preferential activation of hepatic over extrahepatic proteins and the fact that about 50 % of protein S synthesis occurs in endothelial cells imply that the uncarboxylated protein S fraction would also be increased^(6)^. This could increase the risk of thrombosis. Consumption of clotting factors during thrombosis puts a further burden on vitamin K stores by increasing demand for activation of newly synthesised coagulation factors to replace used ones^(55)^. With preference given to the carboxylation of procoagulant factors, progressive depletion of active endothelial protein S increasingly skews the balance towards coagulation.

### Interaction between vitamins D and K

Vitamin D is both endogenously produced in the skin and exogenously acquired from food. Intake of vitamins D and K is correlated due to their co-presence in various food sources. Both vitamin D and K deficiencies are prevalent around the world. Contrary to vitamin K, however, assessment of vitamin D status and propagation of vitamin D supplementation are widespread.

A meta-analysis conducted prior to the emergence of SARS-CoV-2 demonstrated that daily or weekly vitamin D supplementation reduced the risk of acute respiratory tract infection^(56)^. The role of vitamin D in susceptibility to SARS-CoV-2 infection has been assessed by various groups, and to date, results appear to be conflicting^(57–59)^. Studies evaluating the modulatory role of vitamin D on disease severity in Covid-19 have not yet been reported. Vitamin D has anti-inflammatory and anti-proteolytic properties^(60–62)^, which may potentially be favourable in Covid-19. Increasing vitamin D intake is generally regarded to be safe^(63)^, although clinical data are limited. Due to tight hormonal regulation, serum Ca levels are hardly and at most transiently increased even after high-dose vitamin D administration^(64)^. However, short-term hypercalcaemia may induce deposition of Ca on elastic fibres, which is not necessarily released from fibres after normalisation of systemic Ca levels^(64)^. High-dose vitamin D administration in rats depletes extrahepatic vitamin K stores by strongly up-regulating MGP synthesis leading to acceleration of elastic fibre calcification and degradation^(9,64)^. Vitamin D administration in a state of vitamin K deficiency may thereby endanger pulmonary and vascular health. There is also human data that raised these concerns. Vitamin D supplementation was associated with premature mortality in vitamin K-insufficient stable kidney transplant recipients^(65)^. It may therefore be prudent to first supplement vitamin K in invariably vitamin K-insufficient Covid-19 hospitalised patients and to start vitamin D supplementation in those who are vitamin D-deficient only when extrahepatic vitamin K status has been restored^(34)^.

Furthermore, vitamin K might be a useful additive to vitamin D because there is some evidence that it can act as an anti-inflammatory agent by suppressing NF-*κ*B signal transduction. It may also exert a protective effect against oxidative stress by blocking the generation of reactive oxygen species^(66)^.

### Vitamin K antagonists

Although progressively substituted by direct oral anticoagulants, VKA remain important drugs for the prevention of venous and arterial thrombosis. VKA exert their antithrombotic function through inhibition of vitamin K 2,3-epoxide reductase complex 1, thereby interrupting the vitamin K cycle and inducing vitamin K deficiency (Fig. 1). This obstructs carboxylation of hepatic procoagulant factors, which delays blood clotting.

Remarkably, it has been reported that within the epicentre of the Covid-19 outbreak in the UK, the OR of having a supra-therapeutic anticoagulation with VKA (i.e. international normalised ratio > 8·0) was 6·3 around the lockdown date compared with the same period in the year before^(67)^. Root cause analysis suggested that at least 50 % of these elevations were related to Covid-19^(67)^. Although the majority of possible/confirmed Covid-19 cases had used antibiotics which may influence INR^(68)^, we speculate that enhanced pulmonary vitamin K utilisation during SARS-CoV-2 pneumonia could also disturb the narrow therapeutic balance between VKA dosage and vitamin K intake levels.

VKA use in idiopathic pulmonary fibrosis patients associates with reduced survival^(69)^, and it has been suggested that this effect of VKA may be very acute^(70)^. There are reasons to suspect that vitamin K-dependent MGP activation is already compromised in both animals and humans with fibrotic lung disease and that this is further compromised by VKA administration^(71,72)^. Other potential mechanisms by which VKA could exacerbate lung fibrosis may be via preventing anticoagulant protein C and S activation, which both have antifibrotic properties^(73,74)^. Through these mechanisms, VKA may also have an unfavourable effect on pneumonia severity in Covid-19 patients; however, this has not yet been evaluated.

VKA may also have potentially favourable effects on disease course of Covid-19 by prevention of thrombosis, as has been shown for heparins^(75)^. However, considering the consequences of vitamin K insufficiency for pulmonary disease^(69,70)^, it may be worthwhile to conduct a study comparing the risk of severe Covid-19 in patients on VKA with those using other classes of anticoagulant medications, provided the availability of a sufficiently large cohort to correct for confounding factors.

## Future perspective

There is a need for further experimental evidence to link vitamin K deficiency with the pathology of Covid-19 and determine whether vitamin K supplementation has a place in treatment protocols.

First, there is need for lung-specific data. The current data on vitamin K status in Covid-19 are confined to measurement of circulating parameters, and we were unable to distinguish pulmonary from systemic elastic fibre degradation^(34)^. Autopsy studies performed on Covid-19 patients could shed light on the presence of carboxylated and uncarboxylated vitamin K-dependent proteins at sites of SARS-CoV-2-related lung disease. This could give support to our hypothesis of increased pulmonary MGP expression and enhanced vitamin K utilisation. Animal models may also be used to elucidate the effect of vitamin K insufficiency, administration and antagonism specifically on pathologies of pulmonary elastic fibres.

Second, it is important to confirm that protein S activity is decreased during vitamin K insufficiency in Covid-19. This could be explored by measuring protein S activity, but this method has potential confounders^(76)^. An alternative would be to quantify undercarboxylated protein S either with targeted antibodies or using liquid chromatography-tandem MS; however, to our knowledge, such assays have yet to be developed.

Finally, there is need for human intervention studies to determine whether vitamin K supplementation has a place in the prevention and treatment of severe Covid-19. Clinical trials assessing vitamin K administration in hospitalised populations are needed to evaluate both safety and efficacy. The safety of even high doses of vitamin K has been established in healthy persons^(77)^ but remains to be assessed in severely ill Covid-19 patients. The potential role of vitamin K supplementation to prevent development of severe Covid-19 in subjects who have not yet contracted SARS-CoV-2, but are at risk for the infection, is also very relevant to assess.

In conclusion, the potential role of vitamin K supplementation to prevent the development and progression of severe Covid-19 remains largely unexplored. We would argue that the impact of the current crisis warrants thorough evaluation of the therapeutic potential of vitamin K in Covid-19 pathogenesis for two key reasons. Unlike other treatment strategies currently under development for Covid-19 such as dexamethasone, vitamin K does not have any known unfavourable effects in those who do not use VKA. Furthermore, it is relatively simple and inexpensive to manufacture contrary to other therapies like remdesivir or convalescent plasma. Taken together this means that effectiveness can be rapidly and cheaply evaluated in clinical trials and easily implemented if proven successful.
